# Supplementary figures and images for: A clinical score for identifying active tuberculosis while awaiting microbiological results: Development and validation of a multivariable prediction model in sub-Saharan Africa
Source: PLoS Med. 2020 Nov 10;17(11):e1003420. doi: 10.1371/journal.pmed.1003420 (PMC7654801; doi:10.1371/journal.pmed.1003420)

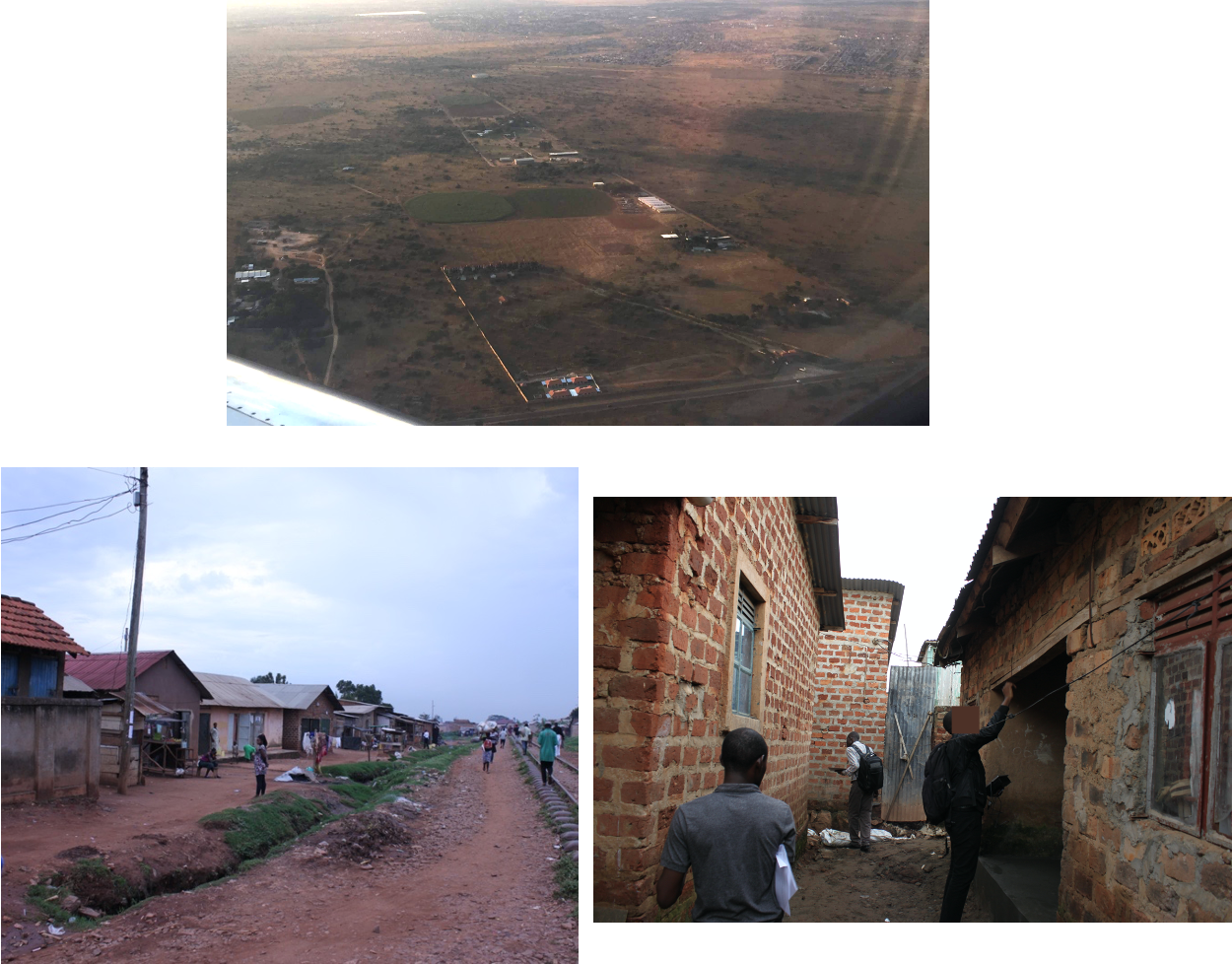

Supplement: S1 Fig — Top is the study site in Limpopo Province, South Africa. Limpopo Province is widely rural and has a low population density (46 people per km2). It has high TB incidence (301 per 100,000) and high adult HIV prevalence (17%). Two photos in the bottom are the study site in the capital city, Kampala, Uganda. In our Ugandan study site, the population density was estimated 23,000 people per km2, TB prevalence was estimated 939 per 100,000 by GeneXpert Ultra catridge (418 per 100,000 after excluding GeneXpert Ultra trace positive but culture negative), and HIV prevalence was 18% among adults. (TIFF) [file pmed.1003420.s002.tiff]

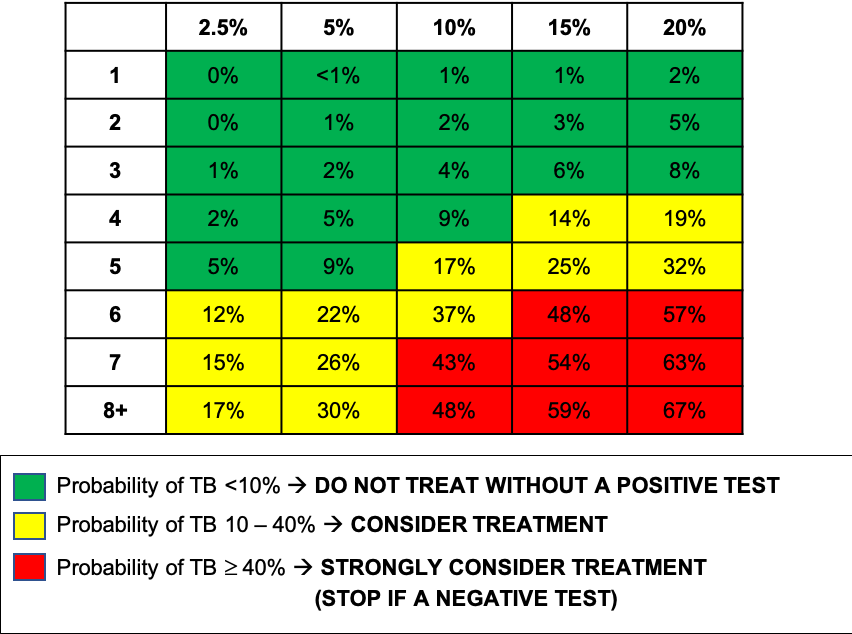

Supplement: S2 Fig — For illustrative purposes, we have chosen cutoffs of 10% and 40% risk of TB as potential clinical decision points, based on natural breaks in predictive probability and on intuition that TB treatment is unlikely to be started empirically for patients with less than a 1 in 10 chance of having TB. Other cutoffs could be selected based on local resources and probabilities of loss to follow-up if untreated. The utility of this score would markedly decrease in settings where the pretest TB probability is below 2.5% because the score higher than 7 is ≥15% at 2.5% TB setting. (TIFF) [file pmed.1003420.s003.tiff]

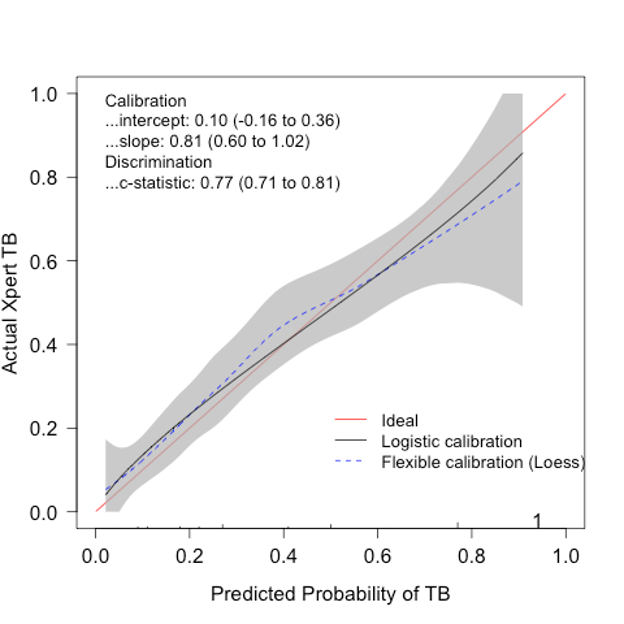

Supplement: S3 Fig — The figure shows model calibration using Cox linear logistic regression in the external validation (Ugandan) population. An intercept of 0 and slope of 1 is consistent with good calibration. In this plot, the red line represents perfect calibration, the black line corresponds to calibration of the simple clinical score, the dotted blue line corresponds to a smoothed (Loess) calibration, and the gray region corresponds to the 95% confidence band. Calibration curves were generated after adjusting for the different sampling fraction of TB in the derivation and validation populations, as described in the main text. (TIFF) [file pmed.1003420.s004.tiff]

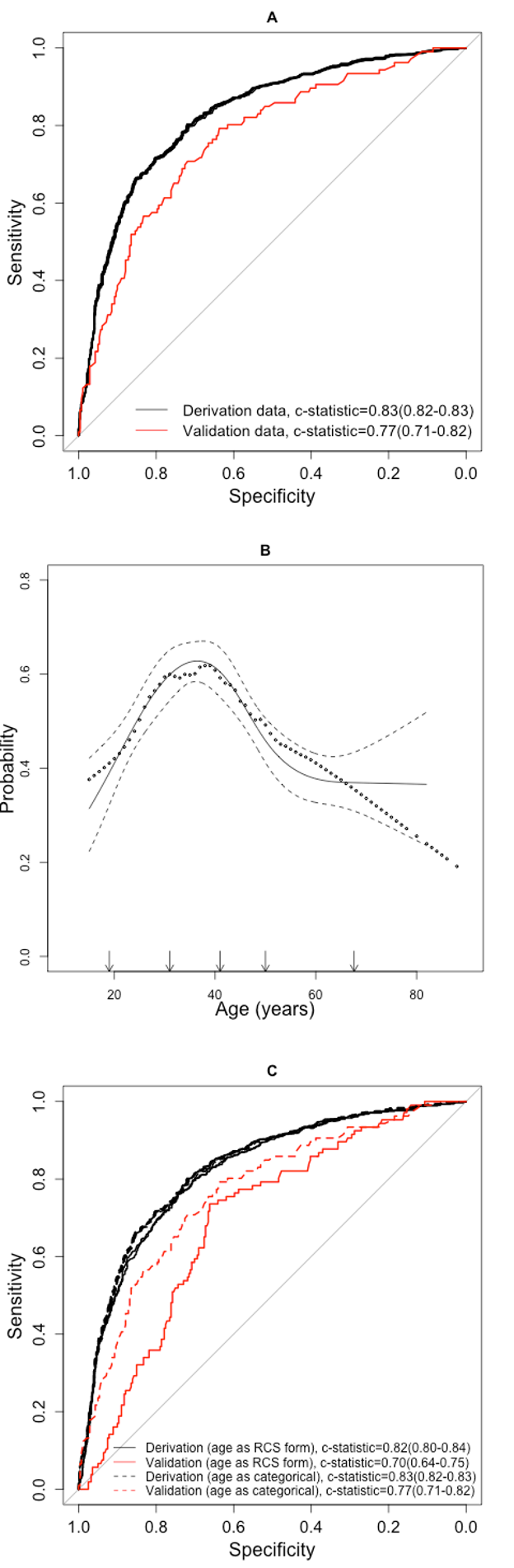

Supplement: S4 Fig — Panel A shows the receiver operating characteristic (ROC) curve—a measure of discrimination—in the South African derivation (black line) and Ugandan external validation (red line) cohorts. The reported c-statistics did not differ with adjustment of the sampling fractions to a population with 10% estimated prevalence. Panel B investigates the nonlinear relationship between the probability of Xpert MTB/RIF result and the continuous variable of age. The R package “Hmisc” was used to plot the estimated restricted cubic splines (RCS; with 5 knots placed at the natural quantiles of age). Arrows on the x-axis show the location of the knots. Of note, the second knot fell very near the median of our highest-risk category (i.e., age 25 to 44 years). Dots show a plot of nonparametric estimates (or smoothing parameters). The solid line shows the estimated spline transformation, and the dotted line shows the 95% confidence lines. Panel C compares the ROC curves using the actual regression coefficients; one with the age modeled as an RCS (solid line) and the other (dotted line) with the age modeled as categorical by decade (i.e., 15 to 24, 25 to 34, 35 to 44, 45 to 54, and 55+). The discrimination of the full model with age as a categorical variable was significantly higher than with age modeled as an RCS but was not statistically different from that of the simple 1 to 10 risk score presented in the main text. (TIFF) [file pmed.1003420.s005.tiff]

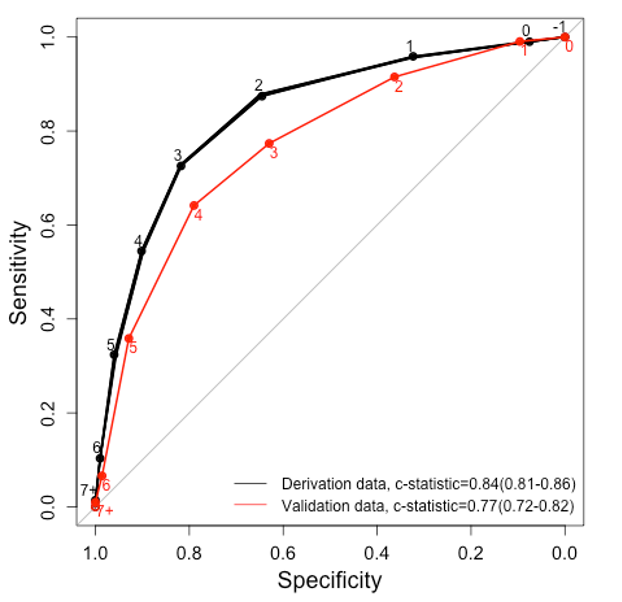

Supplement: S5 Fig — This receiver operating characteristic (ROC) curve shows the discrimination of the model using each of actual classical TB symptoms as individual binary variables, instead of the total number of TB symptoms. The black curves show the clinical score’s discrimination in the derivation (South African) population, and the red curves show the corresponding analysis in the external validation (Ugandan) population. The number on each dot represents the risk score at which sensitivity and specificity are estimated. The discrimination of the model using each symptom individually was not significantly higher than that of the main simple risk score in which symptoms were simply counted, shown in Fig 4B. (TIFF) [file pmed.1003420.s006.tiff]

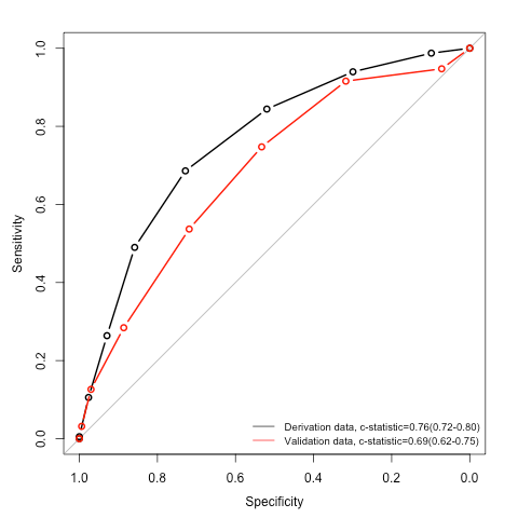

Supplement: S6 Fig — In South African population, 665 (49%) reported chronic (>2weeks) cough, 61% of which were Xpert–positive TB cases in South African population. In Ugandan population, 262 (68%) reported chronic cough in Ugandan population, 36% of which were Xpert–positive TB cases. This receiver operating characteristic (ROC) curve shows the discrimination of the model within individuals who presented chronic cough regardless of any other TB symptoms. The black curves show the clinical score’s discrimination in the derivation (South African) population, and the red curves show the corresponding analysis in the external validation (Ugandan) population. (TIFF) [file pmed.1003420.s007.tiff]

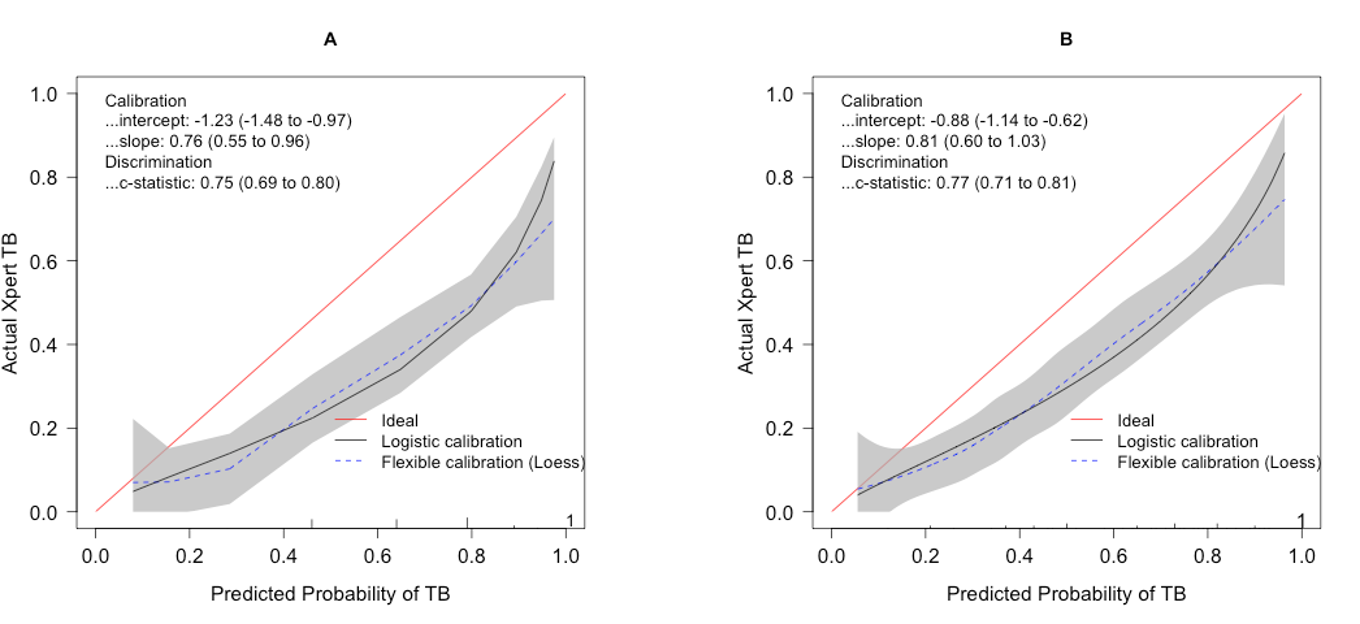

Supplement: S7 Fig — The clinical risk score for active tuberculosis (TB) as derived in the South African population is expected to systematically overestimate the predicted risk of TB in the external validation Ugandan population because of the different sampling fractions used. (Specifically, in South Africa, approximately 1 Xpert-individual was sampled per case, whereas in Uganda, nearly 2.7 Xpert–negative individuals were sampled per case.) To appropriately evaluate model calibration, therefore, we adjusted the intercept of the Cox linear–logistic model by multiplying the log odds ratio of TB in the validation population versus the derivation population. This intercept is a measure of the difference between the predicted probability and the actual outcome (i.e., calibration-in-the-large). Adjusting the intercept (for everyone in the study population) does not affect the order of predicted risks of individuals, and hence, it does not change the predictive accuracy in terms of discrimination (i.e., c-statistics). Panel A shows model calibration using Cox linear logistic regression in the external validation (Ugandan) population before adjusting the intercept, using simple scores as the prediction model. An intercept of 0 and slope of 1 is consistent with good calibration. In this plot, the red line represents perfect calibration, the black line corresponds to calibration of the simple clinical score, the dotted blue line corresponds to a smoothed (Loess) calibration, and the gray region corresponds to the 95% confidence band. The divergence between the black and red lines illustrates the poor calibration prior to adjustment of the intercept; as described above, this poor calibration is expected on the basis of different sampling fractions. Panel A shows calibration of the simple 1 to 10 score, whereas Panel B shows calibration of the model using full regression coefficients. Both of these plots were generated in the external validation (Ugandan) population before adjusting the interce [file pmed.1003420.s008.tiff]

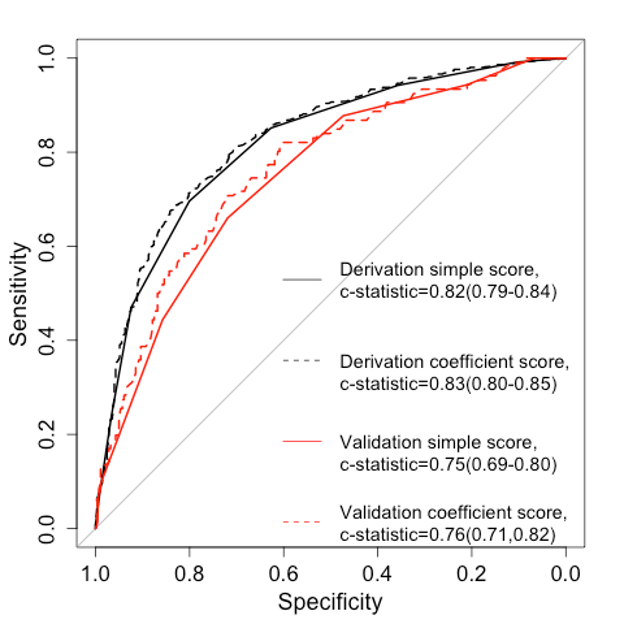

Supplement: S8 Fig — This receiver operating characteristic (ROC) curve shows the discrimination of the model after imputing missing data with the most common value (as might be done in clinical practice), rather than using multiple imputation. The black curves show the clinical score’s discrimination in the derivation (South African) population, and the red curves show the corresponding analysis in the external validation (Ugandan) population. The solid lines and dotted lines show the simple 1-to-10 scoring and scoring system using full regression coefficients, respectively. Comparing this figure to Fig 4B in the main text illustrates that the simple risk score retains the same discrimination (c-statistic 0.75) in the external validation population when using a simple imputation technique (replacing missing values with the most common value in the population), which could be performed easily in clinical practice. (TIFF) [file pmed.1003420.s009.tiff]

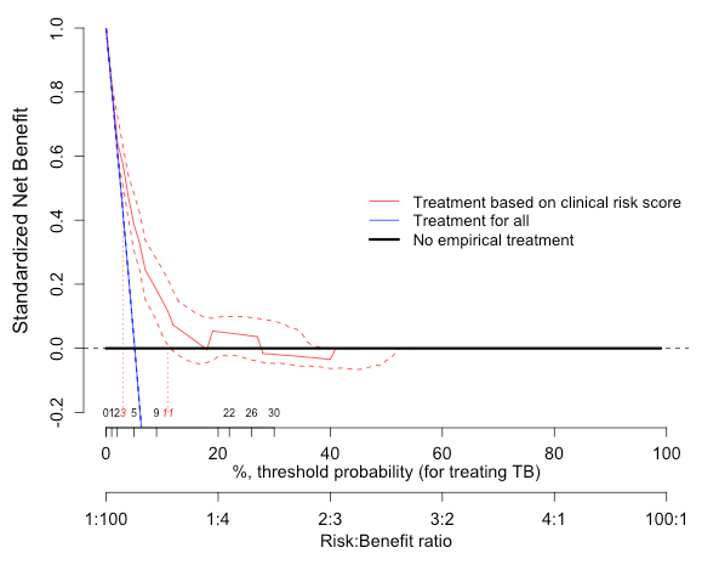

Supplement: S10 Fig — The standardized net benefit (y-axis) was estimated as total benefit (treating true TB) minus total risk (treating false-positive TB), standardized to a maximum benefit of 1, assuming a population with 5% underlying prevalence of TB. The standardized net benefit was examined under different considerations of the relative benefit of a true-positive diagnosis versus the risk of a false-positive diagnosis, or threshold probabilities (x-axis). The decision curve is based on the external validation population, with 95% confidence bands shown as dotted lines. Black numbers on top of the x-axis are the posttest probability we estimated in the main Fig 1 under 5% pretest probability. Red numbers correspond to the threshold probabilities where the lower 95% confidence limit of “treatment based on clinical risk score” line is higher than the upper 95% confidence limit of other lines of 2 different treatment strategies. Use of the clinical risk score of 4 or 5 would offer higher net benefit than alternative treatment strategies (e.g., treatment for all or no empiric treatment) between 3% and 11%. This figure can also be used to evaluate the potential impact of incorrectly assumed pretest probability of TB—for example, if practitioners believe that the pretest probability is 10%, when in reality it is 5%. In this example, the only score in which a misspecification would lead to an incorrect treatment decision is a score of 5. This implies a posttest probability of 17% and an empiric treatment consideration at a 10% pretest probability setting, when in fact the posttest probability is 9% and no empiric treatment consideration required at a 5% pretest probability setting. About 20% of symptomatic individuals may be falsely treated until the microbiological testing confirmation is available. (TIFF) [file pmed.1003420.s011.tiff]

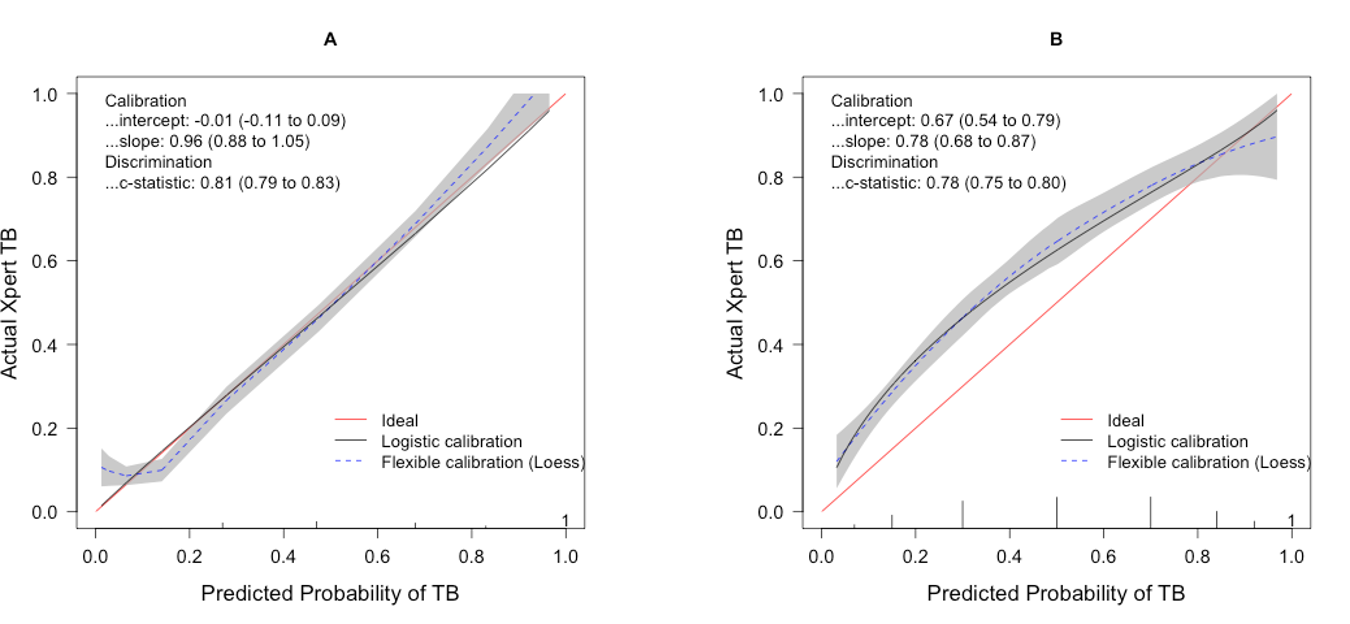

Supplement: S11 Fig — Panel A shows model calibration using Cox linear logistic regression in the internal validation (Ugandan) population, among those whose data did not contribute to model development. An intercept of 0 and slope of 1 is consistent with good calibration. In this plot, the red line represents perfect calibration, the black line corresponds to calibration of the simple clinical score, the dotted blue line corresponds to a smoothed (Loess) calibration, and the gray region corresponds to the 95% confidence band. Panel B shows model calibration in the external validation (South African) population. The calibration curve for Panel B was generated after adjusting for the different sampling fraction of TB in the derivation and validation populations, as described in the text. (TIFF) [file pmed.1003420.s012.tiff]

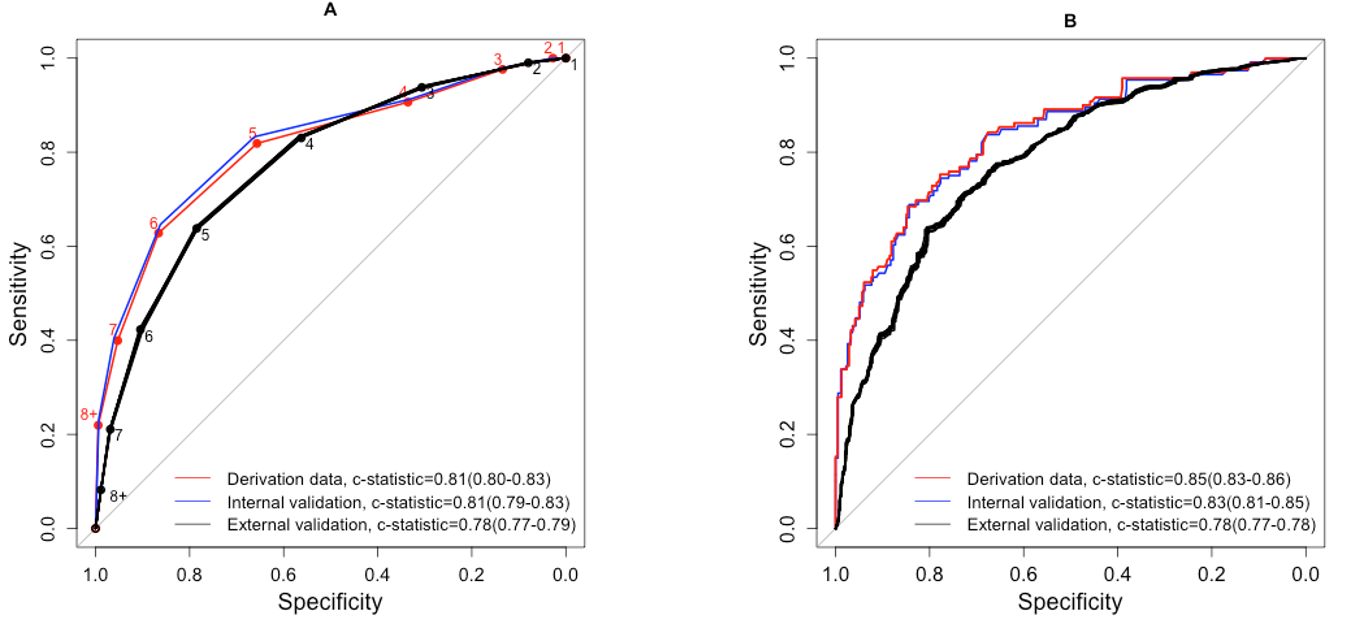

Supplement: S12 Fig — Panel A shows the receiver operating characteristic (ROC) curve in the South African derivation cohort (red line), internal validation cohort (blue line), and Ugandan external validation cohort (black line). The number on each dot represents the risk score at which sensitivity and specificity are estimated. For example, at a score of 5, sensitivity and specificity are 0.66 and 0.82, respectively, in the derivation and internal validation populations, versus 0.79 and 0.64, respectively, in the external validation population. The reported c-statistics did not differ with adjustment of the sampling fractions to a population with 10% estimated prevalence. Panel B shows the ROC curve using more detailed score incorporating full regression coefficients. The discrimination power was retested under an assumption of 10% TB prevalence (by replicating the TB-negative population to fit the target prevalence); the c-statistics remained the same. (TIFF) [file pmed.1003420.s013.tiff]
