# Supplementary material for: A clinical score for identifying active tuberculosis while awaiting microbiological results: Development and validation of a multivariable prediction model in sub-Saharan Africa
Source: PLoS Med. 2020 Nov 10;17(11):e1003420. doi: 10.1371/journal.pmed.1003420 (PMC7654801; doi:10.1371/journal.pmed.1003420)
Supplement: S1 Table — (DOCX) [file pmed.1003420.s014.docx]

## Table S1. Interview questions for self-reported tuberculosis symptoms and HIV status

|  | South Africa study | Uganda study |
| --- | --- | --- |
| TB symptoms | On the day of your clinic visit, which of the following symptoms did you have?   1. Cough 2. Fever 3. Weight loss (more than 5kg or enough to make my clothes loose) 4. Drenching sweats at night | Which of the following symptoms do you have currently? (currently can mean today or within the past few days.) Select all that apply.   1. Cough 2. Coughing up blood 3. Unexplained fever or chills 4. Drenching sweats at night   Within the past twelve months, have you experienced weight loss or more than 5kg or enough to make your clothes loose?   1. Yes 2. No 3. Unknown/refused |
| Length of symptoms | How log had you had that symptom before you came to clinic that day? Response can be in any unit of days, weeks, months, or years. | You said you currently have a cough/coughing up blood/a fever or chills/sweats at night. Now looking back in time, for how long have you had this? (Interviewer should record answer in weeks, round to the nearest week.)  How long ago did you first notice your weight loss? (Interviewer should record answers in weeks, round to the nearest week.) |
| HIV* | (If HIV status based on the clinical register was unknown) Do you know your HIV status, and if you are comfortable to will you please share it with me? | (If HIV lab test result or HIV status based on the clinical register was unknown) Do you have any of the following medical conditions? Select all that apply.   1. Obstructive lung disease (asthma or COPD) 2. Diabetes 3. High blood pressure 4. **HIV or AIDS** 5. Other conditions 6. None |

*We evaluated clinical registers (likely to be available to clinicians) to ascertain HIV status. Rapid HIV testing was offered to all participants, free of charge.
