# Supplementary material for: A clinical score for identifying active tuberculosis while awaiting microbiological results: Development and validation of a multivariable prediction model in sub-Saharan Africa
Source: PLoS Med. 2020 Nov 10;17(11):e1003420. doi: 10.1371/journal.pmed.1003420 (PMC7654801; doi:10.1371/journal.pmed.1003420)
Supplement: S2 Table — The table provides the observed percentage of the population in the external validation population (urban Uganda) who had Xpert-confirmed TB, given each clinical risk score. The observed percentages are compared to the percentages predicted by the clinical risk score (right-most column). The optimal cutoff for clinical decision-making was at a score of ≥4 or ≥5; above these cutoffs, observed and predicted probabilities were similar. (DOCX) [file pmed.1003420.s015.docx]

## Table S2. Observed and predicted probability of active pulmonary TB in the Ugandan external validation population The table provides the observed percentage of the population in the external validation population (urban Uganda) who had Xpert-confirmed TB, given each clinical risk score. The observed percentages are compared to the percentages predicted by the clinical risk score (right-most column). The optimal cutoff for clinical decision-making was at a score of ≥4 or ≥5; above these cutoffs, observed and predicted probabilities were similar.

| **Score** | **Total number of individuals** | **Number of Xpert positive** | **Observed** | **Predicted** |
| --- | --- | --- | --- | --- |
| 1 | 19 | 0 | 0% | 5% |
| 2 | 46 | 6 | 13% | 9% |
| 3 | 81 | 7 | 9% | 16% |
| 4 | 92 | 23 | 25% | 25% |
| 5 | 62 | 23 | 37% | 35% |
| 6 | 52 | 26 | 50% | 43% |
| 7 | 24 | 12 | 50% | 48% |
| ≥8 | 11 | 9 | 82% | 54% |
