# Supplementary material for: A clinical score for identifying active tuberculosis while awaiting microbiological results: Development and validation of a multivariable prediction model in sub-Saharan Africa
Source: PLoS Med. 2020 Nov 10;17(11):e1003420. doi: 10.1371/journal.pmed.1003420 (PMC7654801; doi:10.1371/journal.pmed.1003420)
Supplement: S3 Table — We explored use of the actual regression coefficients from the lasso model (rather than a simple 1-to-10 scoring system), including both a categorical expression of age and a representation of age using restricted cubic splines. For each of these alternative scoring systems, derivation and external validation were performed in the same fashion as for the primary score described in the main text. (DOCX) [file pmed.1003420.s016.docx]

## Table S3. LASSO regression coefficients and the simple scoring system after modeling actual classical TB symptoms as individual binary variables (instead of the total number of TB symptoms) We explored use of the actual regression coefficients from the Lasso model (rather than a simple 1-10 scoring system), including both a categorical expression of age and a representation of age using restricted cubic splines. For each of these alternative scoring systems, derivation and external validation were performed in the same fashion as for the primary score described in the main text.

|  | **LASSO regression**  **Coefficients** | **Score**  ****** |
| --- | --- | --- |
| **Age category, years** |  |  |
| 15 – 24 | 0.49 | 1 |
| 25 – 34 | 0.96 | 1 |
| 35 – 44 | 0.56 | 1 |
| 45 – 54 | 0.25 |  |
| ≥ 55 | Reference |  |
| **Sex** |  |  |
| Female | Reference |  |
| Male | 0.87 | 1 |
| **HIV status** |  |  |
| HIV negative | Reference |  |
| HIV positive | 1.09 | 1 |
| **Classical TB symptoms** |  |  |
| Cough | -0.41 | -1 |
| Fever | 0.17 | 0 |
| Weight loss | 1.59 | 2 |
| Night sweats | 0.63 | 1 |
| **Total number of classical TB symptoms** |  |  |
| < 3 | Reference |  |
| ≥ 3 | 0.14 | 0 |
| **Duration of TB symptoms** |  |  |
| ≤ 2 weeks | Reference |  |
| > 2 weeks | 0.71 | 1 |
| **Any other non-TB symptoms*** | 0.21 | 0 |
| **Diabetes mellitus** (self-report) | 0.75 | 1 |
| **Previous TB diagnosis** (self-report) | 0.20 | 0 |
| **Smoking history** |  |  |
| Never | Reference |  |
| Ever | -0.23 | 0 |

Abbreviations: HIV, human immunodeficiency virus; TB, tuberculosis

* Participants were asked about chest pain, pain elsewhere, skin symptoms, genitourinary symptoms, gastrointestinal symptoms, and “any other symptoms”

** An assigned point for each predictor that is used in the multivariable logistic regression and the LASSO regression. Each point is estimated by dividing each of the LASSO coefficients by the clustered value of the coefficients (to maximize simplicity of the scoring system) and rounding to the nearest integer
