# Supplementary material for: A clinical score for identifying active tuberculosis while awaiting microbiological results: Development and validation of a multivariable prediction model in sub-Saharan Africa
Source: PLoS Med. 2020 Nov 10;17(11):e1003420. doi: 10.1371/journal.pmed.1003420 (PMC7654801; doi:10.1371/journal.pmed.1003420)
Supplement: S4 Table — For the clinical risk score to be useful as a tool in the field, clinicians must be able to directly impute missing data when those data are unavailable. We therefore performed a secondary analysis in which all missing values were assigned the most common value (or single imputation) to reflect likely clinical use of the score in the field. For example, when a clinician does not have information about duration of TB symptoms, the clinician can use the dominant value, 0 or 1, among patients. In our study, a missing duration of TB symptoms, any other non-TB symptoms, and diabetes mellitus was replaced to 1, 1, and 0. After replacing the missing values with the most common value of each variable, the simple score rule based on the least absolute shrinkage and selection operator (lasso) regression coefficients remained the same. We then compared the discrimination of this model using simple (clinical) imputation versus multiple imputation. (DOCX) [file pmed.1003420.s017.docx]

## Table S4. LASSO regression coefficient and simple scoring systems, using a single imputation method (i.e. mode) for the missing predictors For the clinical risk score to be useful as a tool in the field, clinicians must be able to directly impute missing data when those data are unavailable. We therefore performed a secondary analysis in which all missing values were assigned the most common value (or single imputation), to reflect likely clinical use of the score in the field. For example, when a clinician does not have information about duration of TB symptoms, the clinician can use the dominant value, 0 or 1, among patients. In our study, a missing duration of TB symptoms, any other non-TB symptoms, and diabetes mellitus was replaced to 1, 1, and 0. After replacing the missing values with the most common value of each variable, the simple score rule based on the least absolute shrinkage and selection operator (LASSO) regression coefficients remained the same. We then compared the discrimination of this model using simple (clinical) imputation versus multiple imputation.

|  | **LASSO regression**  **Coefficients** | **Score**  ****** |
| --- | --- | --- |
| **Age category, years** |  |  |
| 15 – 24 | 0.40 |  |
| 25 – 34 | 0.93 | 1 |
| 35 – 44 | 0.52 | 1 |
| 45 – 54 | 0.23 |  |
| ≥ 55 | Reference |  |
| **Sex** |  |  |
| Female | Reference |  |
| Male | 0.81 | 1 |
| **HIV status** |  |  |
| HIV negative | Reference |  |
| HIV positive | 1.22 | 2 |
| **Total number of classical TB symptoms** |  |  |
| 1 | Reference | 1 (0) |
| 2 | 0.74 | 2 (1) |
| 3 | 1.76 | 3 (2) |
| 4 | 2.37 | 4 (3) |
| **Duration of TB symptoms** |  |  |
| ≤ 2 weeks | Reference |  |
| > 2 weeks | 0.81 | 1 |
| **Any other non-TB symptoms*** | 0.25 | 0 |
| **Diabetes mellitus** (self-report) | 0.74 | 1 |
| **Previous TB diagnosis** (self-report) | 0.14 | 0 |
| **Smoking history** |  |  |
| Never | Reference |  |
| Ever | -0.25 | 0 |

Abbreviations: HIV, human immunodeficiency virus; TB, tuberculosis

* Participants were asked about chest pain, pain elsewhere, skin symptoms, genitourinary symptoms, gastrointestinal symptoms, and “any other symptoms”

** An assigned point for each predictor that is used in the multivariable logistic regression and the LASSO regression. Each point is estimated by dividing each of the LASSO coefficients by the clustered value of the coefficients (to maximize simplicity of the scoring system) and rounding to the nearest integer
