# Supplementary material for: A clinical score for identifying active tuberculosis while awaiting microbiological results: Development and validation of a multivariable prediction model in sub-Saharan Africa
Source: PLoS Med. 2020 Nov 10;17(11):e1003420. doi: 10.1371/journal.pmed.1003420 (PMC7654801; doi:10.1371/journal.pmed.1003420)
Supplement: S5 Table — To further assess the transportability of the components of the clinical risk score, we performed a de novo model development using the population from Kampala, Uganda, as the derivation population and the population from South Africa as the external validation population. For this analysis, we used a 20-fold bootstrap sample of the Ugandan population (given its smaller sample size) to optimize statistical power. Then, we applied a split internal validation approach by randomly selecting two-thirds of the population as the model training cohort and internally validating the model on the remaining one-third of the testing population (whose data did not contribute to model development). (DOCX) [file pmed.1003420.s018.docx]

## Table S5. Additional characteristics of the model derivation population (Uganda) after bootstrapping and random splitting of the model derivation and the internal validation population To further assess the transportability of the components of the clinical risk score, we performed a *de novo* model development using the population from Kampala, Uganda, as the derivation population and the population from South Africa as the external validation population. For this analysis, we used a 20-fold bootstrap sample of the Ugandan population (given its smaller sample size) to optimize statistical power. Then we applied a split internal validation approach by randomly selecting two-thirds of the population as the model training cohort and internally validating the model on the remaining one-third of the testing population (whose data did not contribute to model development).

|  | **Derivation population** | | **Internal validation population** | |
| --- | --- | --- | --- | --- |
|  | **Xpert positive N=1433 (%)** | **Xpert negative N=3727 (%)** | **Xpert positive N=713 (%)** | **Xpert negative N=1867 (%)** |
| **Coughing observed during interview*** |  |  |  |  |
| None | 812 (57) | 3209 (86) | 419 (59) | 1594 (85) |
| More than once | 621 (43) | 518 (14) | 294 (41) | 273 (15) |
| **Self-reported depression history** |  |  |  |  |
| No | 969 (68) | 2799 (75) | 493 (69) | 1400 (75) |
| Yes | 464 (32) | 928 (25) | 220 (31) | 467 (25) |
| **Involuntarily skipped meals in last month** |  |  |  |  |
| No | 881 (62) | 2133 (57) | 436 (61) | 1037 (56) |
| Yes | 552 (39) | 1594 (43) | 277 (39) | 830 (45) |
| **Household TB contact**** |  |  |  |  |
| No | 1167 (81) | 2989 (80) | 554 (78) | 1511 (81) |
| Yes | 266 (19) | 738 (20) | 159 (22) | 356 (19) |

Abbreviations: HIV, human immunodeficiency virus; TB, tuberculosis

* Coughing observed during interview was reported by the study staff

** Participants were asked if they shared a house or room with the known TB patients
