# Supplementary material for: A clinical score for identifying active tuberculosis while awaiting microbiological results: Development and validation of a multivariable prediction model in sub-Saharan Africa
Source: PLoS Med. 2020 Nov 10;17(11):e1003420. doi: 10.1371/journal.pmed.1003420 (PMC7654801; doi:10.1371/journal.pmed.1003420)
Supplement: S6 Table — (DOCX) [file pmed.1003420.s019.docx]

## Table S6. Association of key variables with Xpert-confirmed pulmonary tuberculosis in the bootstrapped Ugandan population.

|  | **Univariable**  **Odds Ratio^a^ (95%CI)** | **Multivariable**  **Odds Ratio^b^ (95%CI)** | **LASSO regression**  **Coefficients** | **Score^c^** |
| --- | --- | --- | --- | --- |
| **Age category, years** |  |  |  |  |
| 15 – 24 | 1.28 (0.59,2.75) | 2.85 (1.01,7.98) | 1.00 | 1 |
| 25 – 34 | 2.03 (1.02,4.05) | 3.08 (1.25,7.63) | 1.06 | 1 |
| 35 – 44 | 1.83 (0.89,3.78) | 2.68 (1.06,6.80) | 0.98 | 1 |
| ≥ 45 | Reference | Reference | Reference |  |
| **Sex** |  |  |  |  |
| Female | Reference | Reference | Reference |  |
| Male | 2.4 (1.51,3.81) | 2.61 (1.36,5.01) | 0.89 | 1 |
| **HIV status** |  |  |  |  |
| HIV negative | Reference | - | - |  |
| HIV positive | 2.4 (1.51,3.81) | - | - |  |
| **HIV status** |  |  |  |  |
| HIV negative | Reference | Reference | Reference |  |
| HIV positive, on antiretroviral therapy | 0.81 (0.48,1.36) | 1.12 (0.56,2.25) | 0.01 |  |
| HIV positive, not on antiretroviral therapy^d^ | 8.29 (2.59,26.59) | 8.69 (1.83,41.32) | 1.97 | 1 |
| **Classical TB symptoms** |  |  |  |  |
| Cough | 0.75 (0.07,8.39) | - | - |  |
| Fever | 2.54 (1.59,4.08) | - | - |  |
| Weight loss | 4.96 (3.04,8.09) | - | - |  |
| Night sweats | 3.44 (2.08,5.69) | - | - |  |
| **Total number of classical TB symptoms** |  |  |  |  |
| 1 | Reference | Reference | Reference | 1 |
| 2 | 2.27 (1.22,4.2) | 1.76 (0.85,3.65) | 0.49 | 2(0) |
| 3 | 5.37 (2.75,10.5) | 4.21 (1.77,10.00) | 1.30 | 3(1) |
| 4 | 14.84 (6.31,34.86) | 8.45 (2.86,24.95) | 2.03 | 4(2) |
| **Duration of TB symptoms** |  |  |  |  |
| ≤ 2 weeks | Reference | Reference | Reference |  |
| > 2 weeks | 6.46 (3.23,12.92) | 4.59 (2.07,10.19) | 1.45 | 1 |
| **Any other non-TB symptoms^e^** | 2.16 (1.37,3.4) | 1.02 (0.54,1.90) | 0 | 0 |
| **Self-reported comorbidities** |  |  |  |  |
| Diabetes mellitus | 1.33 (0.12,14.81) | - | - |  |
| Obstructive pulmonary disease | 0.37 (0.05,3.07) | - | - |  |
| **Previous TB diagnosis** (self-report) | 2.28 (1.27,4.09) | 2.06 (0.92,4.63) | 0.67 | 0 |
| **Education** |  |  |  |  |
| High school or less | Reference | Reference | Reference |  |
| Any post-high school education | 1.05 (0.66,1.69) | 1.09 (0.58,2.04) | 0.21 | 0 |
| **Smoking history** |  |  |  |  |
| Never | Reference | Reference | Reference |  |
| Ever | 2.34 (1.43,3.82) | 1.88 (0.94,3.73) | 0.71 | 0 |
| **Coughing observed during interview^f^** |  |  |  |  |
| None | Reference | Reference | Reference |  |
| More than once | 4.9 (2.93,8.2) | 6.43 (3.26,12.66) | 1.81 | 1 |
| **Self-reported depression history** |  |  |  |  |
| No | Reference | Reference | Reference |  |
| Yes | 1.54 (0.95,2.52) | 0.55 (0.27,1.10) | -0.61 | 0 |
| **Involuntarily skipped meals in last month** |  |  |  |  |
| No | Reference | - | - | - |
| Yes | 0.81 (0.51,1.29) | - | - | - |
| **Household TB contact^g^** |  |  |  |  |
| No | Reference | Reference | Reference |  |
| Yes | 1.08 (0.62,1.87) | 0.81 (0.38,1.70) | -0.17 | 0 |

Abbreviations: 95%CI, 95% confidence interval; HIV, human immunodeficiency virus; TB, tuberculosis

^a^ Estimated from univariate logistic regression

^b^ Estimated from the multivariable logistic regression, adjusting for all other variables with a population prevalence of at least 10% and a statistically significant association with tuberculosis on univariate regression. Individual TB symptoms were removed in favor of total number of symptoms based on an *a priori* decision.

^c^ Each point in this simple clinical score is estimated by dividing the corresponding LASSO coefficient by the median value of six clustered coefficients (1.4) and rounding to the nearest integer. One point was added to the score for number of TB symptoms to increase usability, as all participants had at least one symptom.

^d^ This category includes HIV positive with unknown antiretroviral therapy status

^e^ Participants were asked about chest pain, pain elsewhere, skin symptoms, genitourinary symptoms, gastrointestinal symptoms, and “any other symptom.”

^f^ Coughing observed during interview was reported by the study staff

^g^ Participants were asked if they shared a house or room with the known TB patients
