# Supplementary material for: A clinical score for identifying active tuberculosis while awaiting microbiological results: Development and validation of a multivariable prediction model in sub-Saharan Africa
Source: PLoS Med. 2020 Nov 10;17(11):e1003420. doi: 10.1371/journal.pmed.1003420 (PMC7654801; doi:10.1371/journal.pmed.1003420)
Supplement: Attachment — Submitted filename: PLOS Medicine review TW 20200525 2pm.docx [file pmed.1003420.s020.docx]

Dr Tom Wingfield MbChB(Hons) PhD DTMH MRCP DipHIV DELE

Consultant Physician and Senior Clinical Lecturer in Infection

Departments of Clinical Sciences and International Public Health

Liverpool School of Tropical Medicine

Email: tom.wingfield@lstmed.ac.uk

25^th^ May 2020

**Re: Review of PMEDICINE-D-20-01816R1 manuscript “A simple clinical score for predicting active tuberculosis when same-day microbiological testing is unavailable”**

Dear Editorial Team of PLOS Medicine,

Thank you for considering me to review this manuscript for PLOS Medicine, which I recommend for publication. The research describes the development and validation of a simple score to predict TB disease in two HIV- and TB-endemic sub-Saharan African countries. The manuscript is important and well planned, executed and written. The findings will be useful to clinicians, researchers, and policy makers, and the methods could be used in other settings to develop similar locally-relevant and context-specific scores (e.g. for places with low HIV burden).

The comments below are minor and I do not need to see a further version of the manuscript (which I believe is already a revised version but I was not involved in the original manuscript review) prior to its publication.

I congratulate the authors on their valuable manuscript and look forward to seeing it in print in PLOS Medicine.

Yours sincerely,

Dr T Wingfield

**Minor Comments**

1. Please can the authors clarify how the random sample of Xpert-negative participants were selected.
2. Given the significant global proportion of people with extra-pulmonary TB, please can the authors consider (perhaps in the discussion and/or limitations section) whether their methods are relevant to - or could potentially be applied to - this group of people.
3. Very minor but can rounding make percentages up to 100 rather than 101 percent in HIV section of GeneXpert positive South African derivation population. Thanks.
4. I note that education level seems to be the only proxy indicator of poverty level. Please can the authors just clarify if other proxy indicators of socioeconomic position were considered and clarify if education level was chosen, as I imagine, to ensure the utility and simplicity of the score for use in clinics.
5. With regards to malnutrition, please can the authors elaborate on the specific reason it was not included in their model (e.g. did not add predictive accuracy or just not collected). Do the authors feel that a subjective assessment of “underweight / normal weight / overweight” by the person performing the score could potentially have utility?
6. It would be good if the authors were able to include some reference to the use of such scores to predict TB in contacts or households affected by TB (<https://www.thelancet.com/journals/laninf/article/PIIS1473-3099(17)30447-4/fulltext> and

<https://www.thelancet.com/journals/laninf/article/PIIS1473-3099(19)30423-2/fulltext>)

7. Could the authors, in a sentence in the discussion or limitations section, mention the possible issue that such a score could potentially reduce the likelihood of a clinician/health care professional sending a diagnostic test (e.g. “they are high risk, the score recommends treating, so why waste resources on sending a sample….)
