# Supplementary material for: A clinical score for identifying active tuberculosis while awaiting microbiological results: Development and validation of a multivariable prediction model in sub-Saharan Africa
Source: PLoS Med. 2020 Nov 10;17(11):e1003420. doi: 10.1371/journal.pmed.1003420 (PMC7654801; doi:10.1371/journal.pmed.1003420)
Supplement: Attachment — Submitted filename: 20200601_PLOS_riskscore_review.docx [file pmed.1003420.s021.docx]

Dr. Matthew Saunders, Clinical Research Fellow

Department of Infectious Disease, Faculty of Medicine

Imperial College London, Hammersmith Hospital Campus

150 Du Cane Road, London W12 0NN, UK

1^st^ June 2020

**Manuscript review: A simple clinical score for predicting active tuberculosis when same-day microbiological testing is unavailable**

Dear Helen Howard and the editorial team at PLOS Medicine,

Thank you for inviting me to review this manuscript, which makes an important contribution to the literature. Please find attached my complete review.

Yours truly,

Dr. Matthew Saunders MRCP(UK) PhD(c) DTMH

**Comments to the authors**

I enjoyed reading this manuscript, which makes an important and novel contribution to the literature. The study aims to address the critical question of how to manage patients presenting to primary care settings with TB symptoms for whom same day microbiological testing is unavailable. This is a thorough and impressive piece of work, with sound statistical methods that are strengthened significantly by the extensive additional analyses presented in the supporting information. Some of the issues I would have highlighted have already been addressed by the authors in these supplementary analyses or have been thoughtfully discussed in the limitations. However, I have a number of comments and suggestions that may help to further strengthen the manuscript that mostly relate to the clinical utility of the score.

**Cut offs and implications for practice.** The authors present standard parameters used to evaluate diagnostic tests. However, I think that what the use of this score in practice might explicitly mean should be more clearly demonstrated, and the implications of specific strategies further considered. There is a slight contradiction in the manuscript because in the risk score figure, the authors are careful to frame clinical recommendations as “consider or strongly consider treatment”. However, in the discussion, the authors highlight that in many resource-constrained settings mid-level clinicians utilize standardised approaches to care. If a standardised approach were to be used, using a score cut off of four for giving empirical TB treatment gives a sensitivity of 85% and a specificity of 63% (optimistic scenario using the derivation data). Translating these figures into a population of 1000 people where prevalence of TB in tested individuals is 10% means that 85 would be appropriately treated for TB, 15 would not be treated immediately despite having TB, and a further 333 would also be given TB treatment who subsequently have a negative Xpert result. Using a different cut-off point would mean different results and this trade-off between sensitivity and specificity is very important and could be further elaborated upon. If the score were to be applied in this standardised way with a cut off of four, this would mean a very large number of people starting TB treatment empirically who subsequently have a negative Xpert result (four times the number starting TB treatment who subsequently have a positive Xpert result). Some of these “negative but treated” people will truly actually have TB, as a single Xpert test on sputum has imperfect sensitivity for pulmonary TB and is inadequate to diagnose extra-pulmonary TB. However, in most cases people who tested negative for TB will truly not have TB and may have an alternative diagnosis such as a simple upper respiratory tract infection. Although the vision is that clinicians will be able to de-escalate treatment, I’m not sure this is likely to happen in practice and feel that once a decision to start TB treatment has been made, it is infrequent for that decision to be reversed, especially in the absence of microbiological/radiological testing. Furthermore, by the time clinicians evaluate people with a negative result to decide about TB treatment de-escalation, a simple upper respiratory tract infection might have spontaneously resolved, appearing to mimic a good response to TB treatment and increasing the chances of the clinician simply continuing TB treatment, inappropriately. An alternative approach of only treating people empirically who have very high-risk scores has its own limitations and I think that these complex trade-off issues merit further discussion in the manuscript.

**Loss to follow-up.** As the authors state, the score is not designed and should not be used to replace definitive microbiological testing, but rather aims to identify people who are at high risk of TB to facilitate empirical management with the aim of reducing loss to follow-up for the highest-risk patients. Therefore, I think the utility of this score depends both on the risk of TB, and on the risk of loss to follow-up for that individual person. Perhaps the authors could discuss in further detail some of the reasons why people are lost to follow-up (e.g. long travel times, costs, stigma) and the potential for derivation of similar algorithms to predict loss to follow-up that could be used alongside their tool to inform optimal decision making. They might also reflect upon other strategies that could be used to reduce pre-treatment loss to follow-up such as incentives and social support interventions. Overall, I think that there is a clear need for more research to address these important questions because the decision to start empirical treatment versus waiting for results/illness evolution depends on many factors, including local epidemiology, severity of illness, availability of further testing/chest radiography, and risk of loss to follow-up.

**Decision curve analysis.** The use of a decision curve analysis is appropriate and interesting. However, I think that people inherently find the interpretation of these analyses and graphics confusing. I think the authors have done a good job in trying to explain and provide context to the findings, but I wonder if they might be able to go even further to make these findings more accessible to people not familiar with the analysis strategy. I also wonder whether “cost:benefit” should be rephrased as “risk:benefit”. Obviously a false-positive diagnosis incurs economic costs to both the person and health system, but also more broader risks associated with inappropriate treatment, adverse drug reactions, and failure to diagnose alternative pathologies. As highlighted above, perhaps some of these trade-off issues could be further explored in the discussion. I was also unclear what statistically significant means in the context of the decision curve analysis, and whether 95% confidence intervals are necessary.

**Minor comments.**

1. Apologies if I have missed it but it wasn’t clear to me what symptoms triggered TB testing in the derivation cohort, and how this differed from how presumptive TB was defined to trigger TB testing in the validation cohort? Table 1 seems to suggest that people had to have at least one of cough, fever, weight loss, or night sweats to receive an Xpert test? Were the same criteria used in both cohorts? In some settings where this score might be most valuable, patients only receive a TB diagnostic test if they have productive cough for over two weeks. Could the authors potentially comment upon how this might affect the interpretation and use of their score?
2. Please provide more detail on the symptoms included in the model. Should clinicians ask about any cough of any duration, or specifically productive cough? Does weight loss have to be over a specified amount or is it simply self-reported? Did the authors have data available on haemoptysis? Do symptoms refer to a certain time period, i.e. if you have had one episode of fever three weeks ago but none since, does this count as having presented with fever?
3. Relatedly, how variables are measured is likely to impact on predictive performance. Diabetes and HIV are self-reported, but these diseases are substantially underdiagnosed in many of the settings where the authors propose the score could be used. I note that HIV status is known for all participants in this study, was that because rapid testing was done at recruitment and so clinicians had up to date results at the time of risk score assessment? What should be done in settings where somebody last had an HIV test three years ago and the assessing clinician does not have access to rapid testing now? Do we assume they are negative, potentially misclassifying them and underestimating their risk of TB? This is complex as in some sub-Saharan African countries HIV prevalence is extremely high, especially in people presenting to healthcare with symptoms. Similarly for diabetes.
4. I think Table 1 should be checked and include missing data for each variable. Some of the numbers don’t add up, e.g. duration of TB symptoms 206+465=671. But n=707 for the column.
5. I think the number 1634 (total population in derivation cohort) is an error in the results. Also, the authors first report non-participation in the discussion. I think a flow chart demonstrating study design and recruitment would significantly add to the manuscript and help readers to understand what the authors did. Relatedly I would be interested to know what the prevalence of Xpert confirmed TB was in people presenting with symptoms at these clinics, to have a clearer understanding of what sampling fraction the authors recruited. How were the Xpert negative people randomly selected? These data and questions could all be easily presented and addressed in a figure.
6. I think a great strength of this study is the derivation and validation of the score in two diverse cohorts. Perhaps the authors could supplement the above suggested figure with some population-level data demonstrating the key differences between these two populations e.g. underlying HIV prevalence, poverty estimates. Photos may also be a nice addition.
7. I was surprised to see that both smoking and previous TB were associated with TB diagnosis in univariable analysis (as you would expect), but not in multivariable analysis. Could the authors comment upon which factors included in the final model are felt to be confounding or mediating these associations?
8. I agree with the importance of deriving a model that could be easily used in a resource-constrained primary health care clinic. However, I can think of a number of variables that might predict TB that have not been included, which would be easily measurable. For example, epidemiological factors such as if the person was known to have a recent TB contact. Relatedly, I think that although BMI might be difficult to measure in many settings, clinicians can easily visually estimate whether a person is underweight versus normal weight versus overweight. If BMI data are available to analyse then I would be interested to see if a variable of weight category predicts TB in these populations, and importantly whether it adds any predictive power to their model.
9. I think the discrimination of the score in the derivation cohort should be mentioned in the main text of the results and not just the figure.
10. Are the sensitivity and specificity in figure 4 based on the performance of the score in the derivation cohort, or the validation cohort? I suspect derivation cohort from studying the data.
11. I think the last sentence of the first paragraph of the discussion should be reworded, emphasising the need for further operational/impact research to investigate the use of a score-based strategy. Further validation alone will not demonstrate that the score can reduce loss to follow-up, transmission, and mortality.
12. I would be interested to know if smear microscopy was available in these clinics and if the authors have data on this? This is still the most commonly used method to diagnose TB globally and is available in many of the settings where this score might be most useful.
13. I think the sentence “These sensitivity analyses showed no material change in discrimination….” would be more appropriate in the results rather than the methods.

None of these comments should take away from the excellent and comprehensive work the authors have done. Please accept my apologies for the rather long review.

Open review

Signed: Matthew Saunders
