# Supplementary material for: A clinical score for identifying active tuberculosis while awaiting microbiological results: Development and validation of a multivariable prediction model in sub-Saharan Africa
Source: PLoS Med. 2020 Nov 10;17(11):e1003420. doi: 10.1371/journal.pmed.1003420 (PMC7654801; doi:10.1371/journal.pmed.1003420)
Supplement: Attachment — Submitted filename: Letter to reviewers_final.docx [file pmed.1003420.s022.docx]

Yeonsoo Baik

Postdoctoral fellow

Johns Hopkins School of Public Health

615 N Wolfe St, Suite E7133

Baltimore, MD 21205

[ybaik3@jhmi.edu](mailto:ybaik3@jhmi.edu)

September 28^th^, 2020

Dr. Clare Stone

Chief Editor

[cstone@plos.org](mailto:cstone@plos.org)

+44 (0)1223 442 818

**Re-submission of manuscript entitled “A simple clinical score for predicting active tuberculosis when same-day microbiological testing is unavailable: a multivariable prediction model using data from a clinical trial in South Africa and a population-based survey in Uganda" (PMEDICINE-D-20-01816R2)**

Dear Dr. Stone and editors:

Thank you so much for accepting our paper. Please see our point-by-point response to the comments below.

------------------------------------------------------------

Requests from Editors:

1- Thank you for editing the title, please revise further to: “A clinical score for identifying active tuberculosis without microbiological testing: development and validation of a multivariable prediction model”

**Thank you for the suggestion. We are somewhat concerned that this suggested title would implicitly encourage use of this clinical score instead of microbiological testing. To make it clear that the score should not replace microbiological testing (and to be responsive to prior comments that we identify the setting), we have revised the title as “A clinical score for identifying active tuberculosis *while awaiting* microbiological results: development and validation of a multivariable prediction model *in sub-Saharan Africa*”. Ultimately, we will defer to the Editors’ judgment, but we do think it important not to suggest in the title that this score should replace the results of microbiological testing.**

2- Second point of Author Summary, “*require* data…”

**We edited this typo.**

3- Thank you for including the TRIPOD checklist. Please add the following statement, or similar, near the start of the Methods: "This study is reported as per the Transparent reporting of a multivariable prediction model for individual prognosis or diagnosis (TRIPOD) guideline developing, validating, or updating a prediction model (S1 Checklist). Please also replace the page numbers in the checklist with paragraph numbers per section (e.g. "Methods, paragraph 1"), since the page numbers of the final published paper may be different from the page numbers in the current manuscript.

**We added the suggested statement, and the page numbers in the checklist were replaced with the paragraph numbers.**

4- Thank you for noting your data are available. At this time, please update your data statement to provide all the information necessary for researchers to access this dataset (eg, accession number, url or email address to contact if an application is required).

**We have updated the data statement (final sentence under “Analysis Plan”) with URLs that link to both a deidentified dataset sufficient to replicate all analyses and a Github repository with our model code. [We are working with the JHU Data Repository to create a URL for a deidentified dataset sufficient to replicate all analyses and will update this as soon as it is available – likely within a week. In the interim, we wanted to resubmit this manuscript within the deadline so as not to slow the publication process.]**

5- Please add a bit more context of why the study is important to the Abstract Background (i.e. note the limited availability of same-day microbiological testing an the risk of losing patients to follow-up).

**We edited the Abstract Background as follows: “In highly resource-limited settings, many clinics lack same-day microbiological testing for active tuberculosis (TB). In these contexts, risk of pre-treatment loss to follow-up is high, and a simple, easy-to-use clinical risk score could be useful..”**

6- In the Abstract Methods and Findings, please include the dates of the two cohorts, and some relevant demographic information (e.g., age, sex, HIV positivity).

**We added the dates of the two cohorts in lines 31 and 36-37 and demographic information in lines 40-42.**

7- The sentence on lines 44-46 needs a bit more context for what the scores of 3 and 7 mean.

**We have updated our description of the score itself to provide this context: “The final prediction model, scored from 0 to 10, included six characteristics: age, sex, HIV (two points), diabetes, number of classical TB symptoms (cough, fever, weight loss, night sweats; one point each), and >14-day symptom duration.” We then refer to the scores as “3/10” and “7/10” rather than “three” and “seven” to help further clarify.**

8- Thank you for adding the limitations of the score on lines 46-47, please also include limitations of the study itself, though.

**We added one major limitation that our study may be subject to spectrum bias as we only included a random sample of people without TB from each cohort.**

9- Similar to the Abstract Background section, the Abstract Conclusions could use a bit more context as well. Please address the study implications without overreaching what can be concluded from the data; the phrase "In this study, we observed ..." may be useful.

**In response to this comment and the comment below, we revised our Abstract Conclusion to read, “In this study, we observed that a simple clinical risk score reasonably distinguished individuals with and without TB among those submitting sputum for diagnosis. Subject to prospective validation, this score might be useful in settings with constrained diagnostic resources where concern for pre-treatment loss to follow-up is high.”**

10- Around line 51, you could add "subject to prospective validation" or similar

**Thank you for this suggestion, which we have incorporated as above.**

11- Please move the reference brackets in front of punctuation. E.g.: “... the leading single-agent cause of infectious mortality worldwide [1].”

**We moved the reference brackets as indicated through the manuscript.**

12- Table 1: please make clear that the numbers in the parentheses are percentages. Also, “irregular” is misspelt.

**We added “N (%)” to the table heading and fixed the spelling error.**

13- Table 2: please include p-values alongside the 95% CIs.

**We created a new column in the Table 2 and added p-values.**

14- The numbers above the x-axis on figure 4 are a bit difficult to interpret. Please consider reformatting the “Score” and “Post-test probability” labels with smaller fonts and re-aligning. Perhaps the scales below could be smaller to allow more space?

**We reformatted the x-axis to be read easily and edited the figure caption accordingly.**

15- Please use the "Vancouver" style for reference formatting, and see our website for other reference guidelines

> Reference 7, 14, 15 may need reformatting.

> For reference 17, 18, please provide the date accessed.

> Missing information from Reference 31?

**We reviewed and edited the reference formatting.**

16- Figure S1 could be moved to the main text. The background renders in dark grey on my computer, which makes the arrows difficult to see.

**We removed the grey background from the figure, and now the previous Figure S1 is the main Fig 1.**

17- The third image from Figure S2 may need to be edited to blur the face of the person on the right. I think he’s probably identifiable.

**We blurred the face of the person in S1 Fig (previous Figure S2).**

18- References to the “Appendix” throughout the the main text should be changed to reference the relevant supplementary file(s).

**We indicated the specific supplementary file number accordingly.**

19- Please remove the funding information from the "Acknowledgements" at the end.

**The funding information is now removed from the Acknowledgements.**

Comments from Reviewers:

Reviewer #4

4. Single imputation values might be derived from Table 1 on hindsight, which might or might not be further emphasized.

**We also have presented the single imputation values as a footnote under the table embedded in Figure 2.**

12. Additional caveats have been described discussion appropriate adjustments, were microbiological results not as expected according to the assumed TB probabilities. Ideally, the effect of incorrect TB probability assumptions might be more quantitatively explored.

**We added quantitative explanations in the caption for S10 Fig, where we replicated the decision curve analysis in a different TB prevalence setting.**

Production Issues:

FIGURES and GRAPHICS:

1. Please ensure that all main figures are referenced in the manuscript as Fig 1, Fig 2, etc. (including capitalization), rather than Figure 1, Figure 2, etc. Please note, however, that the file names themselves must not include the space, i.e. Fig1.tif, Fig2.tif. Please ensure that all main figure files are clearly labeled as such in the File Inventory.

**We referenced the figure numbers named the figure files as suggested.**

METADATA:

1. Please accurately complete the 'Competing Interests' section on our submission form, including any COIs declared by your co-authors. If you have no competing interests to declare, please add "The authors have declared that no competing interests exist". Otherwise please declare all competing interests beginning with the statement "I have read the journal's policy and the authors of this manuscript have the following competing interests:"

As one of your authors has declared COI, please use the second statement.

2. Your co-author, Dr. Martinson, has declared the following competing interest in relation to this manuscript. Please include it in the main competing interest statement in our system, sticking as closely as possible to Dr. Martinson's wording, using only their initials.

"My institution receives a grant from Pfizer to follow up patients with pneumonia."

**We have updated the COI.**

3. Please ensure only the authors' initials are included in the competing interest statement, not their full names or titles.

**We have updated the COI only with the authors’ initials.**

4. Please provide a detailed Financial Disclosure statement. This is published with the article, therefore should be completed in full sentences and contain the exact wording you wish to be published.

- Please clarify all sources of funding (financial or material support) for your study. List the grants (with grant number) or organizations (with url) that supported your study, including funding received from your institution.

- State the initials, alongside each funding source, of each author to receive each grant. [NOTE: Please do NOT include full author names or titles.]

- State what role the funders took in the study. If the funders had no role in your study, please state: “The funders had no role in study design, data collection and analysis, decision to publish, or preparation of the manuscript.”

- If any authors received a salary from any of your funders, please state which authors and which funders.

If you did not receive any funding for this study, please state: “The authors received no specific funding for this work.”

**We have our current Financial Disclosure statement as “**

**The study was funded by the US National Institutes of Health (R01AI116787 and R01HL138728) to David W Dowdy (DWD).
The funders had no role in the study design, data collection and analysis, decision to publish, or preparation of the manuscript.”**

4. In accordance with PLOS' data policy, please ensure that your Data Availability Statement in the submission form clearly identifies how readers can access your data. Note that it will be typeset as it is written so please ensure it is in complete sentences and appears as you would like it to in the published version.

**We have updated the data statement (final sentence under “Analysis Plan”) with URLs that link to both a deidentified dataset sufficient to replicate all analyses and a Github repository with our model code. [We are working with the JHU Data Repository to create a URL for a deidentified dataset sufficient to replicate all analyses and will update this as soon as it is available – likely within a week. In the interim, we wanted to resubmit this manuscript within the deadline so as not to slow the publication process.]**

ARTICLE FILE:

1. Please indicate the corresponding author on the title page with an asterisk (*) and include the corresponding author's email address. Only an asterisk and the email address itself are required.

Please format as follows, omitting all author name, title, address or contact information:

* [ybaik3@jhmi.edu](mailto:ybaik3@jhmi.edu)

**We have only left an asterisk and the email address for the corresponding author information.**

2. Please remove author degree titles from the byline of your manuscript file.

**We removed author degree titles.**

3. Please ensure that all authors have at least one affiliation listed on the title page and that all listed affiliations are attributed to at least one author. Affiliations should be listed in ascending numerical order in the affiliation list and should match what is provided in the Authors Information in the submission form.

Affiliations should include an institution, a city, and a country (no postcode). US affiliations need the state as well, written out in full (with no abbreviations).

**We reviewed the affiliations.**

4. Please ensure that your Ethics Statement is available in the Methods section of your manuscript in its entirety, under the subheading "Ethics Statement".

**Ethical consideration was included in the Methods section. We have updated the subheading as Ethics Statement at line 240.**

5. PLOS does not publish Appendices. Please incorporate them into the main manuscript, or upload each of your Appendices as a Supporting Information file in the form "S1 Text", “S2 Text” etc, and refer to it throughout the manuscript as such.

**We have uploaded each Appendix as a Supporting Information file in the form suggested.**

ACKNOWLEDGMENTS:

1. Please remove the funding information from the Acknowledgments section of your manuscript file. The published version is pulled automatically from the “Financial Disclosure” field in our submission form and must not be duplicated. Please therefore take this opportunity to check that the information in this field is correct and completed in full.

**The funding information is now removed from the Acknowledgements.**

SUPPORTING INFORMATION:

1. Please confirm that either you are the photographer of Figure S2, or that you will provide written permission from the photographer to publish them under a CC-BY license.

**We have provided written permission from the photographers to publish them under a CC-BY license.**

2. Figure S2 includes an image of an identifiable person or persons. Please provide written confirmation or release forms, signed by the subject(s) (or their guardian), giving permission to be photographed and to have their images published under a creative commons license.

Otherwise, we kindly request that you remove the photograph.

**We have blurred the face of the person in S1 Fig (previous Figure S2).**

3. Please ensure that all Supporting Information files use the following format verbatim throughout the manuscript (including legends and all in-text citations): S1 Fig, S1 Table, S1 Text, S1 Data, etc. Each file type should start at 1 and increase numerically. Please note, however, that the file names themselves must use an underscore rather than a space, i.e. S1_Fig.tif, S1_Table.xlsx, S1_Text.doc, S1_Data.xlsx, S1_PRISMA_Checklist.docx, etc.

**We changed the supporting information legends, in-text citations, and file names as suggested.**

4. Please note that your submission cannot contain any references to generic "supporting information" files and instead must refer to specific files using our naming convention (S1 Fig [not S1 Figure/Figure S1/Fig S1], S1 Table, S1 Text, S1 Data, etc.)

**We edited and referred to specific file numbers ad format.**

5. Your Tripod Checklist will need to be published with your article. Please therefore ensure that it has the file type 'Supporting Information' rather than file type ‘Other’ in the File Inventory and ensure it adheres to our naming conventions.

**(We now have submitted the TRIPOD Checklist as the Supporting Information)**
